# Biosurfactant from endophytic *Bacillus pumilus* 2A: physicochemical characterization, production and optimization and potential for plant growth promotion

**DOI:** 10.1186/s12934-021-01533-2

**Published:** 2021-02-08

**Authors:** Olga Marchut-Mikołajczyk, Piotr Drożdżyński, Arkadiusz Polewczyk, Wojciech Smułek, Tadeusz Antczak

**Affiliations:** 1 Institute of Molecular and Industrial Biotechnology, Faculty of Biotechnology and Food Science, Lodz University of Technology, Stefanowskiego 4/10, 90-924 Łódź, Poland; 2 Department of Organic Chemistry, Faculty of Chemical Technology, Poznan University of Technology, Berdychowo 4, 60-965 Poznań, Poland; 3 Polytechnic Faculty, Food Technology and Human Nutrition, State University of Applied Sciences, Nowy Świat 4 st., 62-800 Kalisz, Poland

**Keywords:** Food industry wastes, Biosurfactant, Optimization, Endophytes, *Bacillus pumilus* 2A, Plant-growth promotion

## Abstract

**Background:**

Microbial surfactants called biosurfactants, thanks to their high biodegradability, low toxicity and stability can be used not only in bioremediation and oil processing, but also in the food and cosmetic industries, and even in medicine. However, the high production costs of microbial surfactants and low efficiency limit their large-scale production. This requires optimization of management conditions, including the possibility of using waste as a carbon source, such as food processing by-products. This papers describes the production and characterization of the biosurfactant obtained from the endophytic bacterial strain B*acillus pumilus* 2A grown on various by-products of food processing and its potential applications in supporting plant growth. Four different carbon and nitrogen sources, pH, inoculum concentration and temperature were optimized within Taguchi method.

**Results:**

Optimization of bioprocess within Taguchi method and experimental analysis revealed that the optimal conditions for biosurfactant production were brewer’s spent grain (5% w/v), ammonium nitrate (1% w/v), pH of 6, 5% of inoculum, and temperature at 30 °C, leading to 6.8 g/L of biosurfactant. Based on gas chromatography–mass spectrometry and Fourier transform infrared spectroscopy analysis produced biosurfactant was determined as glycolipid. Obtained biosurfactant has shown high and long term thermostability, surface tension of 47.7 mN/m, oil displacement of 8 cm and the emulsion index of 69.11%. The examined glycolipid, used in a concentration of 0.2% significantly enhanced growth of *Phaseolus vulgaris* L. (bean), *Raphanus L.* (radish), *Beta vulgaris* L. (beetroot).

**Conclusions:**

The endophytic *Bacillus pumilus* 2A produce glycolipid biosurfactant with high and long tem thermostability, what makes it useful for many purposes including food processing. The use of brewer’s spent grain as the sole carbon source makes the production of biosurfactants profitable, and from an environmental point of view, it is an environmentally friendly way to remove food processing by products. Glycolipid produced by endophytic *Bacillus pumilus* 2A significantly improve growth of *Phaseolus vulgaris* L. (bean), *Raphanus* L. (radish), *Beta vulgaris* L. (beetroot). Obtained results provide new insight to the possible use of glycolipids as plant growth promoting agents.

## Background

Surfactants are amphiphilic compounds composed of hydrophilic and hydrophobic groups. These molecules can reduce the surface and interfacial tension between liquids, solids and gases. Due to their properties and chemical structure, surfactants are widely used as emulsifiers, detergents, dispersants, semiconductors and wetting agents, which has led to the production of over 15 million tons of surfactants annually [1–5]. As a result of their wide use, surfactants are largely released into the environment, which can cause contamination and pose a threat to all living organisms, including humans. These compounds move along with the movements of air masses over long distances and along with its variable humidity they can settle in soil and water causing their contamination and at the same time threatening penetration into the trophic chain and causing adverse changes. In the aquatic environment, surfactants increase the rate of eutrophication by limiting the concentration of dissolved oxygen. The negative effect of surfactants on humans has been proved—among others endocrine disorders, skin irritation, and also trigger allergies [6, 7].

Microbial surface-active agents called biosurfactants, provide a valuable alternative for synthetic surfactants. These compounds are produced by both bacteria and fungi. Based on the chemical composition, biosurfactants can be classified as lipopeptides and lipoproteins, glycolipids, phospholipids, fatty acids, polysaccharide-lipid complexes and polymeric surfactants [8, 9]. Compared with chemically synthesized surfactants, microbial surface active compounds are characterized by higher biodegradability, lower toxicity as well as better stability and foaming properties in different environmental conditions [8, 10, 11]. These characteristics have led to a growing interest in biosurfactant for use not only in bioremediation and oil processing, but also in the food and cosmetic industries and even in medicine [12]. Furthermore, unlike their chemical counterpart produced based on fossil fuels, biosurfactants can be obtained using waste materials, including agricultural waste. The use of renewable raw materials and fossil resources to produce new products is a pillar of a circular economy. Examples of products based on this type of raw material are biological surface active compounds [5, 13]. Agricultural waste and food processing by-products can serve as a carbon source for the processes of microbial biosurfactant production, due to its availability and low costs (they constitute 30–50% of municipal solid wastes) [13]. Simultaneously, this may generate an environmental friendly method of waste disposal [14]. Production of biosurfactants on cheap agricultural waste (wheat bran, waste cooking oil, grease waste) has been reported [15, 16].

Also, Moshtagh et al. [14] investigated the possibility of biosurfactant production by *Bacillus subtilis* N3-1P using brewery waste. However, high costs of the production of microbial surfactants and low yield limit their large scale production. This requires optimization of culturing conditions and large scale production, including the use of waste as a carbon source [17].

Endophytic microorganisms are bacteria and fungi that live in plant tissues without causing any negative changes in the host's organism (physiological, epidemiological or pathogenic). Endophytic microbes are ubiquitous—they inhabit the tissues of all plant species [18–20]. It is known that endophytic microorganisms can stimulate plant growth, produce biologically active compounds (antibiotics, biosurfactants, phytohormones), increasing the host's resistance to stressful environmental conditions, increasing resistance to pathogens and pests [21].

In our previous work [20], we have described the potential of endophytic *Bacillus pumilus* 2A, isolated from *Chelidonium majus* L. herb, for biosurfactant production. We also showed the positive effect of biosurfactant on plant growth in a polluted environment. The aim of the research was to optimize the production of biosurfactant by endophytic *Bacillus pumilus* 2A with the use of various types of food processing by-products (spent grain, beet pulp, molasses, used cooking oil), characterizing its chemical structure and use as a plant growth promoting agent. In order to obtain the highest possible efficiency of the most stable production of biosurfactants, a series of experiments was conducted based on the Taguchi experiment design.

## Materials and methods

### Biological material

The bacterial endophytic biosurfactant producer *Bacillus pumilus* 2A was previously isolated from *Chelidonium majus* L. herb and deposited in the Institute of Molecular and Industrial Biotechnology collection. Preparation and isolation of the endophytic strain from *Chelidonium majus* L. was carried out according to the previously described procedures [20]. In short, the plant material was carefully excavated from the area adjacent to the A1 motorway near Stryków in Poland and transported to a laboratory in plastic containers, where it was rinsed under running water. Then surface sterilization was performed of healthy plants parts with use of 1% sodium hypochlorite, 70% ethanol and sterile water. Then plants parts were cut into small pieces (~ 1 cm) and placed on the sterile agar NB medium. The plates were incubated at 30 °C for 5 days. The pure colonies were selected, picked up and transferred to slant specific media (LB, Chapek-dox) and screened for degradation and emulsifying activity [20].

After isolation, pure culture of *Bacillus pumilus* 2A was preserved in 20% glycerol at − 80 °C. This endophytic bacteria, among other eleven isolates had the highest emulsifying activity and emulsion index [20].

### Substrate for optimization of biosurfactant production

For optimization of biosurfactant production, four different carbon sources: beet pulp (Total sugars 51.5%; Lipids 8.6%; Protein 25.7%; Mineral elements 3.5%; Ash 2.9% of dry weight) molasses (Lipids 0.7%; Proteins 2.1%; Total sugars 49.5%; Ash 8.5%); brewer’s spent grain (Total sugars 49.2%; Lipids 7.65%; Protein 29.8%; Mineral elements 4.2%; Ash 2.8% of dry weight) and waste cooking oil (Palm oil 50%, Palmitic acid 40%, Oleic acid 10%, contain ω-6 polyunsaturated acids) were used. Beet pulp and molasses were obtained from Polish National Sugar Company factory in Dobrzelin (Poland). Brewery spent grain as a by-product of breweries was donated from SULIMAR Ltd. Company and waste cooking oil was obtained from local restaurants in Lodz, Poland. All of used substrates were stored at 4 °C until needed.

### Inoculum

*Bacillus pumilus* 2A strain was stored on agar slopes made of solid "A" medium with the following composition (g/L): 2.0 glucose, 2.0 yeast extract, 1.5 anhydrous Na_2_HPO_4_, 2.5 NH_4_Cl, 25 agar at − 20 °C [20]. To prepare inoculum one loop of bacterial biomass from slants was suspended in a 500 mL flask containing 40 mL of nutrient broth medium of the following composition: (g/L) 15.0 peptones, 3.0 yeast extract, 6.0 NaCl, 1.0 D( +) glucose. Before sterilization by autoclaving (121̊C, 15 min) pH of the nutrient medium was adjusted to 7.0. The bacteria were cultured in the Infors incubator shaker for 24 h at 30 °C, 180 rpm. After 24 h optical density of the culture media was measured. For the biosurfactant production different concentrations of inoculum (OD_600_ = 0.6) was used.

### Biosurfactant production

Endophytic bacteria were inoculated into 1-L Erlenmeyer flasks containing 350 ml of the mineral medium previously described by Marchut-Mikołajczyk et al. [22]. Biosurfactant production was conducted for 10 days on a rotary shaker (180 rpm), at 30 °C. Different carbon and nitrogen sources (5% w/v and 1% w/v, respectively), pH of medium, temperature and inoculum concentration were used according to the experimental design presented in Tables 1 and 2. Medium without inoculation was used as a negative control.

**Table 1:** Design of experiment with Taguchi method: selected factors and designated levels

Symbol	Factor	Level			
		L1	L2	L3	L4
A	Carbon source	Brewer’s spent grain	Waste cooking oil	Molasses	Beet pulp
B	Nitrogen source	Monosodium glutamate	Ammonium nitrate	Ammonium sulfate	Corn soak
C	pH	5	6	7	8
D	Inoculum (%)	3	5	7	9
E	Temperature (°C)	20	30	37	45

**Table 2:** Design summary—Taguchi orthogonal array table of Lg (5^4^) for experimental conditions

Run	Carbon source	Nitrogen source	pH	Inoculum (%)	Temperature (°C)
1	L3	L1	L3	L4	L2
2	L1	L1	L1	L1	L1
3	L2	L2	L1	L4	L3
4	L1	L2	L2	L2	L2
5	L4	L1	L4	L2	L3
6	L3	L3	L1	L2	L4
7	L4	L2	L3	L1	L4
8	L1	L3	L3	L3	L3
9	L2	L4	L3	L2	L1
10	L3	L2	L4	L3	L1
11	L1	L4	L4	L4	L4
12	L4	L4	L1	L3	L2
13	L3	L4	L2	L1	L3
14	L2	L1	L2	L3	L4
15	L4	L3	L2	L4	L1
16	L2	L3	L4	L1	L2

### Design of experiments

In order to optimize the process of biosurfactant production by *Bacillus pumilus* 2A Taguchi method was used. A L16 orthogonal array composed of 16 experimental setups was used. We have investigated the effect of five factors and their impacts in four different levels, (Tables 1, 2). In order to reduce experimental errors each experiment was repeated three times.

The indexes of 1, 2, 3 and 4 indicate the levels of the factors, while symbol L is the abbreviation of level. The samples were prepared according to the orthogonal array of L16 conditions. The aim of optimization was to select such levels of input factors that would ensure the highest amount of effective and stable biosurfactant. Therefore the response of the system was defined as emulsifying activity (OD_500_), emulsion index (IE24), and the amount of biosurfactant. Experiments were repeated three times for each setup to avoid systematic errors. Parameters with the highest desired value were adopted and the S/N ratio (controllable factors/confounding factors, ETA) was calculated using the formula:

$$(S/N)_{HB} = - 10\log \left[ {\left( \frac{1}{n} \right)\sum {\left( {\frac{1}{{y_{i}^{2} }}} \right)} } \right]$$

where i—number of measurements, n—number of measurements for a specific measurement, y—measured feature.

### Biosurfactant isolation and purification

In order to produce biosurfactant, the culture broth was centrifuged (10000 rpm, 4 °C, 20 min). Obtained supernatant was acidified with 6 M HCl to pH 2 and left overnight in refrigerator (4 °C). Then the liquid was centrifuged again at the conditions mentioned above. Supernatant was discarded and the precipitate was dissolved in 0.1 M NaHCO_3_ and lyophilized. Then lyophilized samples were extracted overnight with chloroform and methanol mixture (2:1). The solvent mixture was evaporated in a vacuum evaporator, and the extracted biosurfactant was collected [20, 23]. To assess the best conditions for biosurfactant production by *Bacillus pumilus* 2A, obtained extracted biosurfactants were weighed. The yield of biosurfactant production was calculated by dividing the obtained mass of the dried product by the total volume of the crude biosurfactant solution.

### Characterization of biosurfactant

Carbohydrate and protein content was evaluated with phenol and sulphuric acid method and Bradford method, respectively [24, 25]. Fourier transform infrared spectrometry analysis was performed on a Thermo Scientific NICOLET 6700 FTIR spectrophotometer to detect the nature of the biosurfactant obtained under the optimized conditions. Spectra were analyzed in transmittance mode within the wavelength range of 4000–400 cm^−1^ [22]. Temperature stability of obtained biosurfactant was carried out in range of temperatures of 30–100 °C [12].

### Composition of biosurfactant assessment by GC/MS analysis

Analytical grade N,O-Bis trifluoroacetamide (BSTFA) and acetic anhydride were purchased from Sigma-Aldrich, Poland. Other chemicals used, like methanol and hexane, were purchased from POCH Avantor, Poland. To 100 mg of each sample, 0.1 mL of derivatization reagent BSTFA was added and mixed. After incubation at 60 °C for 30 min, the sample was dissolved in 0.5 mL of hexane. The second series of the samples were analyzed via transmethylation with method described by Sasser [26]. The third derivatization method used was acetylation with 0.2 mL acetic anhydrous. The incubation conditions were as for BSTFA derivatization.

PEGASUS 4D GCxGC-TOFMS gas chromatograph (LECO Corp., St. Joseph, MI, USA) connected to a BPX5 (5% phenyl equivalent, 28 m × 0.25 mm; 0.25 μm) capillary column (SGE Int., Melbourne, Australia) was used for qualitative analysis. Conditions of the GC–MS analysis: Helium as carrier gas (flow of 1.0 mL/min); ion source and transfer line temperature − 250 °C; splitless injection; sample volume 1 μL; temperature program: oven temperature – 40 to − 300 °C at a rate of 12 °C/min, the oven temperature maintained for 15 min. The acquisition rate was set at 10 spectra/s.

### Emulsifying activity (OD_500_) and emulsion index IE24

To evaluate the emulsification capacity of the produced biosurfactant the emulsifying activity and emulsifying index IE24 were measured [27]. 2 ml of crude biosurfactant solution was added into test tubes containing 2 ml of diesel oil. The mixture was vortexed vigorously (2 min) and left undisturbed for 24 h. The emulsion index (IE24) was calculated by dividing the height of emulsion by the height of whole mixture, multiplying by 100 [14].

### Oil displacement test

Oil displacement test was conducted according slightly modified Morikawa method [28]. 1 mL of mineral oil on the surface of 100 mL of water in a Petri dish with diameter of 15 cm. Then, the 20 µL of biosurfactant solution was delicately applied on the oil drop. The established clean zone was measured 30 s after the application of the biosurfactant solution compared with 1 ml of distilled water as a negative control [29, 30].

### Surface tension analysis

The interface properties of the biosurfactant was evaluated by measuring the equilibrium surface tension of the extract solutions at 21 ± 1 °C using the du Nouy platinum ring technique with Easy Dyne K20 tensiometer (Krüss, Germany). The biosurfactant solutions were prepared using the deionised and ultrapurified Mili-Q water (18.2MΩ m).

### Thermostability

The stability in various temperature variants (30, 37, 55, 75 and 100 °C) was determined by preparing mixture of 2 ml of oil and 2 ml of 2 and 3% biosurfactant solutions and incubating them at the above-mentioned temperatures for 15 min. After this time, the emulsion index was determined according to “Emulsifying activity (OD500) and emulsion index IE24” section.

### Effect of biosurfactant from endophytic *B. pumilus* 2A on plant growth

The seeds *Phaseolus vulgaris* L. (bean), *Raphanus* L. (radish), *Beta vulgaris* L. (beetroot) were surface sterilized with 1% (w/v) sodium hypochlorite followed by three washings with sterile water. Ten sterilized seeds of each plant were placed on a cotton wool moistened with 20 mL of crude biosurfactant solution at concentration of 0.2 and 0.4% in a plastic beaker and incubated for 5 days at 25 °C under a 12 h dark / 12 h light photoperiod. After this time the mass of grown plants was measured. Reference samples contained sterilized seeds incubated on moistened with sterile water cotton wool, incubated in the same conditions as described above.

### Data analysis

Statistica 10.0 program was used for the calculation of mean values, standard deviations and the analysis of variance (single factor ANOVA). Analyses were carried out in triplicate. Tukey’s test was used to test the differences between results represented as individual means and control mean ± standard deviation. Significance was set at p = 0.05 and *p*-values ≤ 0.05 were considered significant.

## Results and discussion

### Determination of optimum conditions for biosurfactant production by endophytic *B. pumilus* 2A

Primary optimum conditions for the production of surface-active compounds by endophytic bacteria *Bacillus pumilus* 2A, isolated from *Chelidonium majus* L. were found using Taguchi method [20].

**Fig. 1:**
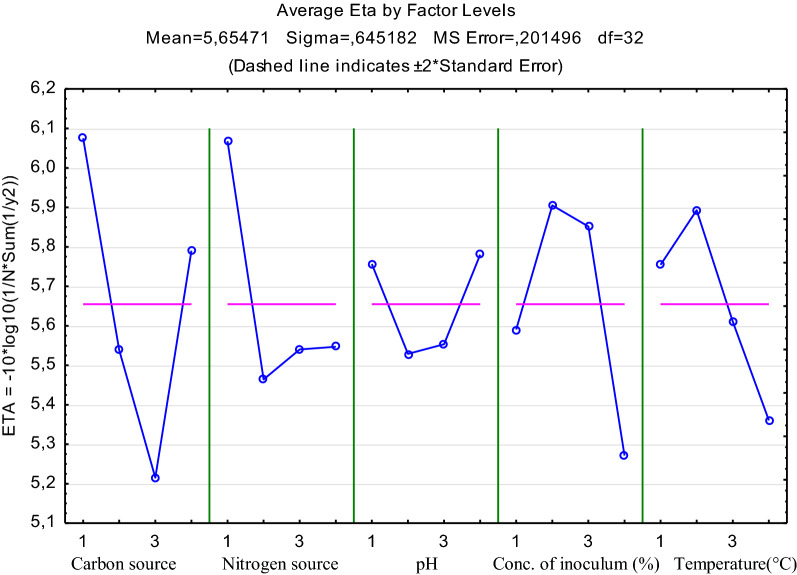
Effect plots of the main factors

Quality characteristic (QC) of bigger-the-best was chosen. The main factor (carbon source, nitrogen source, pH, amount of inoculum, temperature) effect plots are shown in Fig. 1. According to the results, the optimal levels of the tested factors for the production of biosurfactants were: A1B1C4D2E2 (i.e. A at level 1, B at level 1, C at level 4, D at level 2 and E at level 2).Taking into consideration the contribution ratio of each factor it can be concluded that carbon source (42.03%) and nitrogen source (41.30%) have the highest impact on biosurfactant production by *B. pumilus* 2A. pH of the culture broth has the least influence on the process (12.55%).

Nonetheless, some of the obtained results for response variables (Table 3) are not consistent with doses envisioned from Taguchi method (Tables 1 and 2). In terms of the first factor (carbon source) the highest values of examined parameters (emulsifying activity (OD_500_), emulsion index (IE24) and the amount of biosurfactant (g/L) were obtained for brewer’s spent grain, as predicted from the Taguchi method. For this carbon source the yield of biosurfactant production by *B. pumilus* 2A reached 7.125 g/L with simultaneous highest emulsion index (IE24). Furthermore, these results indicate that the production of biosurfactant by endophytic *Bacillus pumilus* 2A on brewery’s spent grain is not only efficient (comparing to other tested substrates) but also that obtained biosurfactant has high emulsifying capacity (IE24 = 43.3%).

**Table 3:** Factors average effects on emulsifying activity (OD_500_), emulsion index (I24) and the amount of biosurfactant

Sample	Emulsifying activity OD_500_	Emulsion index I24 (%)	Amount of biosurfactant (g/L)
1	1.839 ± 0.036	3.51 ± 0.35	3.65 ± 0.43
2	2.145 ± 0.033	43.30 ± 2.41	7.13 ± 0.11
3	1.783 ± 0.012	15.12 ± 0.66	3.25 ± 0.33
4	2.085 ± 0.034	31.50 ± 1.03	6.46 ± 0.18
5	1.824 ± 0.017	17.56 ± 1.19	2.35 ± 0.04
6	1.835 ± 0.033	5.76 ± 0.20	1.97 ± 0.16
7	1.807 ± 0.023	9.52 ± 0.63	2.87 ± 0.11
8	2.004 ± 0.032	34.63 ± 0.38	4.48 ± 0.13
9	1.926 ± 0.043	17.27 ± 0.58	2.96 ± 0.22
10	1.874 ± 0.027	4.17 ± 0.73	1.16 ± 0.09
11	2.052 ± 0.033	26.67 ± 0.93	6.15 ± 0.48
12	1.903 ± 0.056	12.19 ± 1.13	4.98 ± 0.39
13	1.739 ± 0.076	5.49 ± 0.48	2.06 ± 0.28
14	1.934 ± 0.062	16.05 ± 0.94	5.43 ± 0.16
15	1.837 ± 0.039	11.05 ± 0.34	1.56 ± 0.05
16	1.937 ± 0.113	13.64 ± 1.56	3.99 ± 0.04

In terms of nitrogen source, the highest values of examined parameters were obtained for ammonium nitrate, which was compatible with results predicted from Taguchi method. Saikia et al. [29] found that the highest biosurfactant production by *Pseudomonas aeruginosa* RS29 cultivated on glycerol were obtained when ammonium nitrate was used as nitrogen source.

Dikit et al. [31] also showed the highest yield of biosurfactant (4.62 g/L) production by *Agrobacterium rubi* L5 using monosodium glutamate as nitrogen source. However, the authors indicated molasses in the amount of 5.0% (w/v) as the optimal carbon source for biosurfactant production by examined bacteria. The results were similar to those obtained in the present research. While using molasses as the carbon source for *B. pumilus* 2A strain in the same 5% w/v concentration, the maximum yield of biosurfactant was 4.1 g/L. Our results showed that optimization of the carbon source is crucial for microbial production of biosurfactants. Under optimal conditions (using draff (5% w / v) as a carbon source and ammonium nitrate (1% w / v) as nitrogen source), the production efficiency of *B. pumilus* 2A endophytic biosurfactant can reach 7.125 g / l.

As can be seen from the Table 2, in case of pH expected results are inconsistent with the Taguchi experimental design. According to Taguchi method the highest values of emulsifying activity, emulsion index and the amount of obtained biosurfactant should have been obtained for pH 9. However, high values of examined parameters were obtained also for pH 3, 5 and 7, depending on other investigated factors, like carbon and nitrogen source, inoculum concentration and temperature. The obtained results suggest that the pH of the culture broth does not have a significant influence on the biosurfactant production process by *B. pumilus* 2A.

Additionally, the results obtained for tested inoculum concentrations were consistent with those predicted from Taguchi method. As expected, the highest values of examined parameters were obtained for 5% of inoculum. Our results are consistent with those obtained by Fouda et al. [32], who examined seven different concentrations of inoculum 0.5, 1, 2, 4, 6, 8, and 10% (v/v) to optimize biosurfactant production by *Pseudomonas aeruginosa* 4.2 and *Bacillus cereus* 2.3 bacteria strains. Their results showed that the inoculum of 4–6% led to maximum production rates.

The highest values of emulsifying activity, emulsion index and the amount of biosurfactant were obtained when the process was carried out at the temperature of 30 °C, which was supported by the Taguchi method. According to Bertrand et al. [33] the highest yield of biosurfactant production for *Bacillus mycoides* and *Bacillus brevis* strains may be obtained in the range of 35–40 °C However, the temperature of the bioprocess is closely related to the production costs. The results presented in this report show that the production of biosurfactant by *B. pumilus* 2A at 30 °C can take place at a lower temperature, which can lead to lower production costs.

However, Sahoo et al. [34] found that the temperature of 30 °C is the most suitable for the production of biosurfactants by *Pseudomonas aeruginosa* OCD1. Also, Najafi et al. [35] reported that the temperature of 30 °C is optimal for the production of biosurfactants by *Bacillus mycoides*. These data correspond with our results placed in Table 3.

### Isolation and purification of biosurfactant in optimal conditions

As mentioned before, according to the result obtained from the Taguchi method carbon source has the highest impact (42.03%) on biosurfactant production by *B. pumilus* 2A. At the same time, ammonium nitrate as a nitrogen source, pH of 6, 5% w/v of inoculum and temperature of 30 °C turn out to be optimal for the process. Therefore, biosurfactant production was carried out using different carbon source as the only variable.

The bacterial growth, amount of obtained biosurfactant, emulsifying activity, emulsion index and oil displacement activity were analyzed (Table 4). The highest amount of biosurfactant (6.8 g/L) was obtained for the brewer’s spent grain used as a sole carbon source. Furthermore, in this variant of the process the highest growth of *Bacillus pumilus* 2A was observed. The use of brewer’s spent grain as a carbon source resulted in the highest emulsifying activity (OD_500_ 2.07 ± 0.069) and emulsion index (IE24 57.14 ± 0.007%). Moreover, for this variant of experiment the highest oil displacement activity was noted. Moshtagh et al. [14] reported that the brewery waste in the concentration of 7% w/v can be used for biosurfactant production by *Bacillus subtilis* N3-1P.

**Table 4:** Summary of the biosurfactant production by *B. pumilus* 2A strain on different waste from food industry

Carbon source	Biosurfactant amount (mg/L)	Bacterial biomass (g/L)	pH post-culture	Emulsifying activity OD_500_	Emulsion index (%)	Oil displacement (cm) ± 2 mm
Molasses	4068.57 ± 81.37	4.042 ± 0.08	6.83 ± 0.2	1.977 ± 0.096	30.77 ± 0.104	5
Beet pulp	650.01 ± 32.50	0.758 ± 0.01	7.00 ± 0.2	1.704 ± 0.181	31.09 ± 0.075	4
Brewer’s spent grains	6800.02 ± 136.0	5.367 ± 0.01	8.20 ± 0.2	2.067 ± 0.069	57.14 ± 0.007	8
Waste cooking oil	641.67 ± 32.08	0.808 ± 0.01	6.13 ± 0.2	1.556 ± 0.151	34.17 ± 0.137	3.5

However, it is difficult to compare their results with data obtained in the present study. Moshtagh et al. [14] used only two response variables (surface tension and emulsification index (IE24) in their optimization experiments and there is no information concerning the amount of obtained surfactant. Fooladi et al. [36] reported biosurfactant production by *Bacillus pumilus* 2IR. The strain was isolated from an oil field. However, on the basis of the amount of obtained biosurfactant (1.08 g/L) authors claimed that investigated strain cannot be consider as good biosurfactant producer. These results are inconsistent with those obtained for endophytic *Bacillus pumilus* 2 A strain. In our experiments utilization of brewer’s spent grain (5% w/v) resulted in the production of high amount of biosurfactant with very good emulsifying (57.14 ± 0.007) and oil displacement activity (8 cm). Therefore, the examines strain can be consider as a good biosurfactant producer. Farhan et al. [17] reported biosurfactant production by *Bacillus sp.* MTCC 5877 cultivated on different carbon sources e.g. glycerol, sodium citrate, glucose. Although, maximum emulsifying activity was high (75%), oil displacement area obtained for the biosurfactant did not exceed 6 cm. Studies on the physicochemical characteristics and stability were carried out using biosurfactant extracted from the culture performed in optimal conditions. Crude biosurfactant was obtained via acid precipitation followed by solvent extraction method.

### Physicochemical characteristics of biosurfactant

#### FTIR analysis

The structure of biosurfactant produced by endophytic *Bacillus pumilus* 2A has been studied by different analytical methods. FTIR technique was used to evaluate the molecular composition of biosurfactant. The absorption bands at 3292.42 cm^−1^ correspond to –OH stretching of carboxylic acid groups. The adsorption peaks at 1642 and 1743 cm^−1^ indicate the C = O stretching and the presence of ester carbonyl group, respectively. The peaks at 1453.40 and 1124.36 cm^−1^ suggest the presence of stretching bands of carbon atoms with hydroxyl groups in the structure of sugar moiety [33]. Furthermore, bands at 1045.92 and 862.03 cm^−1^ was associated with the stretching vibrations of glycosidic linkage [37]. These results confirm that the surface active compound produced by *Bacillus pumilus* 2A belongs to the group of glycolipid biosurfactants.

#### GC–MS analysis

Chromatographic analysis performed after silanization of samples showed that the lipid moiety of *B. pumilus* 2A biosurfactant composed of 9,12-octadecadienoic (linoleic) acid methyl ester. This is the first report showing linoleic acid as the only hydrophobic part of *Bacillus spp.* biosurfactants. Clements et al. (2019) reported conjugated linoleic acid as a novel insecticide against *Leptinotarsa* decemlineata [38]. Due to the content of linolenic acid in the molecule, the biosurfactant produced by *B. pumilus* 2 A may also have potential use as a bioinsecticide*.* However, this phenomenon requires further research*.* Moussa and Azeiz [38] described lipopeptide produced by *Rhodococcus equi* which lipid moiety consisted of 28.7%; palmitic oleic acid, 15.4% 10-methyl stearic, 12.09% 6-octadecenoic acid, and 17.81% linoleic acid. Additionally, small amount of glycerol and monoglycerides were observed. Analysis of the hydrophilic part of biosurfactant revealed a three-sugar glycolipid structure consisting of D-glucose, and D-arabinose and D-xylose.

### Surface tension

**Fig. 2:**
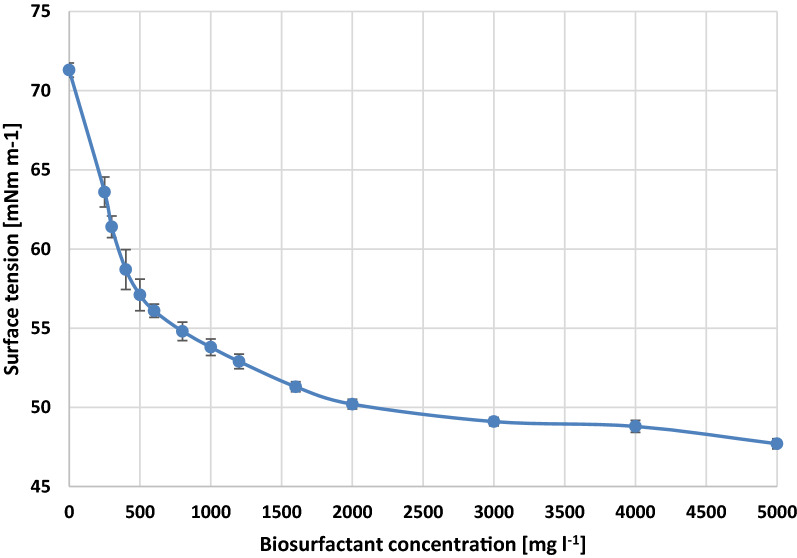
Surface tension at different concentrations of biosurfactant produced by endophytic *Bacillus pumilus* 2A on brewer’s spent grain

The dependence of surface tension and the biosurfactant concentration was presented in Fig. 2. The highest concentration providing homogeneous solution was 5000 mg/L. At this concentration the surface tension decreased to 47.7mN/m. With decreasing concentration the surface tension increased up to 53.8 mN/m at 1000 mg/L and 63.6 mN/m at 250 mg/L.

Similar results were obtained by Oliveira and Garcia-Cruz [39] who studied the biosynthesis of biosurfactant by *Bacillus pumilus* on vinasse and waste frying oil. The best reduction in surface tension of obtained biosurfactant was 45mN/m. Also, Bento et al. [40] obtained 49.5mN/m surface tension for biosurfactant produced by *Bacillus pumilus* on yeast extract.

### Thermostability

After 15 min of incubation samples were cooled at room temperature and the emulsion index (IE24) was measured as an indicator of biosurfactant stability [12].

The effect of temperature on biosurfactant stability revealed that *Bacillus pumilus* 2A produces stable surface active agent in high range of temperature 30–100 °C (Table 5). The emulsion index fluctuates slightly depending on the temperature, although surprisingly its highest values were recorded at 75 °C for 2% solution of biosurfactant. For the higher concentration (3%) of the biosurfactant solution, the highest values of the emulsion index (76.45%) were obtained after 24 h. However, apart from the results obtained at 100° C, only slight differences in the stability of the 2 and 3% biosurfactant solutions were observed. Similar phenomenon was noted by Moussa and Azeiz [39]. They observed only slight fluctuations in the stability of biosurfactants produced by *Rhodococcus equi* and *Bacillus methylothrophicus* strains in the temperature range 20–120 °C. Hatef and Khudeir [41] found during their experiments that the biosurfactant produced by the *Pseudomonas putida* PS6 strain maintains stability in the temperature range of 20–70 °C, although with increasing temperature over 70 °C this stability clearly decreases. The highest stability was noted after 24 h (IE_24_ = 75%) and decreased in time. Nevertheless, even after nineteen days biosurfactant maintain almost 82% of its stability (IE_496_ = 62%). According to Ruggeri et al. [42] the strain can be considered as biosurfactant producer if surface tension is reduced to < 50 mN/m and and/or at least 50% emulsification is observed after 24 h [42]. *Bacillus* *pumilus* 2A cultivated on brewer’s spent grain demonstrated both of those features, thus it suggests that the strain can be used for biosurfactant production.

**Table 5:** Thermostability of biosurfactant solution produced by *Bacillus pumilus* 2A on brewer’s spent grain

Temperature (°C)	Emulsion index IE24		Emulsion index IE48		Emulsion index IE96		Emulsion index IE456	
	Concentration (% w/v)		Concentration (% w/v)		Concentration (% w/v)		Concentration (% w/v)	
	2	3	2	3	2	3	2	3
30	69.62 ± 1.30	66.26 ± 0.91	64.41 ± 1.59	66.11 ± 0.78	65.07 ± 0.61	64.96 ± 0.05	59.94 ± 0.09	56.26 ± 0.93
37	63.26 ± 0.75	69.96 ± 1.68	63.08 ± 0.50	69.59 ± 2.14	62.57 ± 0.09	65.28 ± 1.58	56.29 ± 0.21	62.22 ± 0.61
55	68.02 ± 0.17	76.45 ± 1.22	68.14 ± 0.03	69.52 ± 1.07	63.61 ± 0.50	61.64 ± 1.24	61.73 ± 0.56	57.47 ± 0.68
75	75.51 ± 0.50	66.59 ± 1.29	71.02 ± 1.83	66.62 ± 1.23	67.70 ± 0.79	60.51 ± 1.08	62.74 ± 0.24	55.39 ± 0.72
100	64.00 ± 0.31	57.12 ± 1.36	58.96 ± 0.11	53.64 ± 1.53	46.81 ± 1.02	43.46 ± 1.04	45.68 ± 1.32	41.20 ± 0.64

### Effect of biosurfactant from endophytic *B. pumilus* 2A on plant growth

**Fig. 3:**
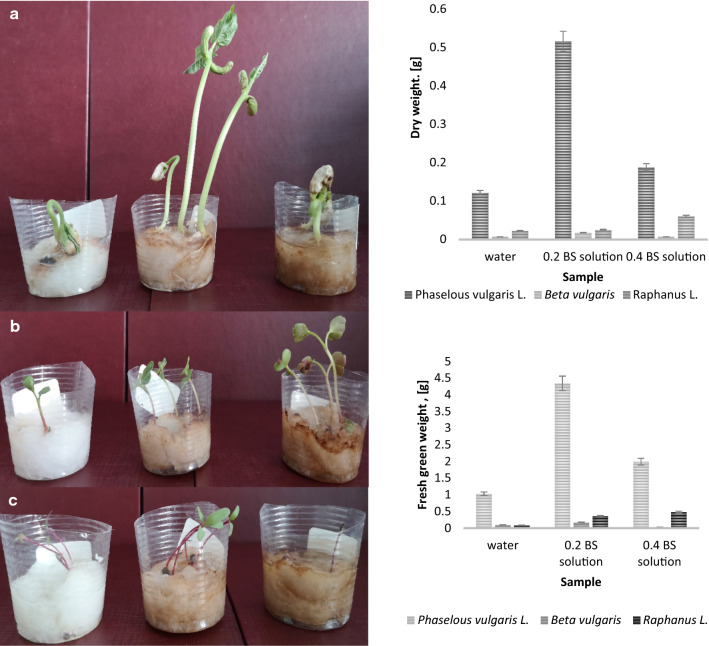
Effect of biosurfactant obtained from endophytic *Bacillus pumilus* 2A cultivated on brewer’s spent grain on plant growth promotion, fresh green weight and dry weight of **a** *Phaselous vulgaris* L. (bean), **b** *Raphanus* L. (radish); **c** *Beta vulgaris* L. (beetroot)

We have previously described the positive impact of biosurfactant produced by *B. pumilus* 2A on the germination and seeding of *Sorghum saccharatum*, *Sinapis alba* and *Lepidium sativum* on soil contaminated with hydrocarbons, using phytotoxicity tests [16]. In this study biosurfactant from *Bacillus pumilus* 2A obtained under optimized conditions was used to investigate its effect on plant growth in in vitro experiments (Fig. 3).The use of biosurfactant solutions resulted in enhanced growth of examined plants. The greatest stimulation of plant growth was obtained for biosurfactant solution used in 0.2% concentration. Comparing to control samples using 0.2% of biosurfactant solution resulted in 4 times, 4 times and 2 times higher growth for bean, radish and beetroot, respectively. For higher concentration of glycolipid lower stimulation of growth was observed. Research on the impact of biosurfactants on plant growth is scarce. It is assumed that microbial surfactants may indirectly promote plant growth by increasing the bioavailability of hydrophobic compounds to microorganisms living in the rhizosphere [20, 43, 44]. The weaker effect of plant growth stimulation observed at a higher concentration of *B. pumilus* 2A biosurfactant may result from an increase in the amount of hydrophobic compounds in the environment, which rhizosphere microorganisms were unable to assimilate, or from the release of compounds adsorbed in the soil, which inhibited the growth of these microorganisms. Also, higher concentration of biosurfactants may cause damage to root plant tissue [45]. Most of the research on the effect of biosurfactants on plant growth concerns environments polluted with hydrocarbons or heavy metals.

Our research shows the possibility of using biological surfactants as ecological, inexpensive and easy to obtain agents that can be used in agriculture to promote plant growth. However, more research is still required to explain the mechanism of the effect of biosurfactants on plant growth.

## Conclusion

To conclude, endophytic *Bacillus pumilus* 2A produce glycolipid biosurfactant with high and long term thermostability, what makes it useful for many purpose including food processing. The use of brewer’s spent grain as the sole carbon source makes the production of biosurfactants profitable, and from an environmental point of view, it is an environmentally friendly way to remove food processing by-products. Glycolipid produced by endophytic *Bacillus pumilus* 2A significantly improve growth of *Phaseolus vulgaris* L. (bean), *Raphanus* L. (radish), *Beta vulgaris* L. (beetroot). Our results provide new insight to the possible use of glycolipids as plant growth promoting agents.

## Data Availability

The data collected upon which this article is based upon are all included in this manuscript.
